# Scoping literature review on the basic health benefit package and its determinant criteria

**DOI:** 10.1186/s12992-018-0345-x

**Published:** 2018-03-02

**Authors:** Ramin Hayati, Peivand Bastani, Mohammad Javad Kabir, Zahra Kavosi, Ghasem Sobhani

**Affiliations:** 1 Student Research Committee, School of Management and Medical Informatics, Shiraz University of Medical Sciences, Shiraz, Iran; 2 Health Human Resources Research Center, School of Management and Medical Informatics, Shiraz University of Medical Sciences, Shiraz, Iran; 3 Health Management and Social Development Research Center, Golestan University of Medical Sciences, Gorgon, Iran; 4 Social Determinants in Health Promotion Research Center, Hormozgan Health Institute, Hormozgan University of Medical Sciences, Bandar Abbas, Iran

**Keywords:** Universal health coverage, Basic health benefit package, Priority setting, Criteria

## Abstract

**Background:**

There are various criteria and methods to develop Basic Health Benefit Package (BHBP) in world health systems. The present study aimed to extract criteria used in health systems in different countries around the world using scoping review method.

**Methods:**

A systematic search was carried out in Cochrane Library, PubMed, Scopus, Science Direct, Web of Science, ProQuest, World Bank, World Health Organization, and Google databases between January and April 2016. Papers and reports were gathered according to selected keywords and were examined by two authors. Finally, the criteria were extracted from the selected papers.

**Results:**

The primary search included 8876 papers. After studying the articles’ titles, abstracts, and full texts, 9 articles and 14 reports were selected for final analysis. After the final analysis, 19 criteria were extracted. Due to diversity of criteria in terms of number and nature, they were divided into three categories. The categories included intervention-related criteria, disease-related criteria, and community-related criteria. The largest number of criteria belonged to the first category. Indeed, the most widely applied criteria included cost-effectiveness (20), effectiveness (19), budget impact (12), equity (12), and burden of disease (10).

**Conclusion:**

According to the results, different criteria were identified in terms of number and nature in developing BHBP in world health systems. It seems that certain criteria, such as cost-effectiveness, effectiveness, budget impact, burden of disease, equity, and necessity, that were most widely utilized in countries under study could be for designing BHBP with regard to social, cultural, and economic considerations.

## Background

Provision of healthcare services at reasonable cost is a worldwide challenge. In this context, numerous reforms were implemented in healthcare systems. Nowadays, health system reforms with focus on Universal Health Coverage (UHC) have been implemented as overall trends in many countries to meet the needs of public health in the context of rapidly changing socioeconomic issues and health resource constraints [1]. The ultimate goal of UHC is access to needed services without the risk of financial hardship and the consequent poverty [2].

Achievement of UHC in many countries will be a long journey along a meandering road. In this trip, every country needs to answer questions, such as which people, what services, and how much cost sharing should be covered. Regarding what services should be covered, evidence has revealed that even countries with the highest health per capita in the world are not capable of covering all services to all people and they need to use Basic Health Benefit Package (BHBP) [3, 4].

BHBP includes all services, procedures, and equipment covered by general state budgets, compulsory insurance programs, or national health services [5]. BHBP is one of the important factors in the implementation of UHC by controlling health costs, assuring services’ quality, and prioritizing health policies [3].

According to World Health Organization’s (WHO) report, three prerequisites have been mentioned on the path to UHC. One of these prerequisites needs efficient and fair spending of money. BHBP could be used as one of the methods to improve efficiency and equity [6].

BHBP significantly includes different interventions in different countries, which reflects diverse economic, epidemiological, and social conditions. For example, in low-income countries, BHBP covers a limited list of public health and clinical services provided at first and second levels. On the other hand, in richer countries, this package is designed as what services could not be listed in the package [4]. In addition, there are different views regarding the development of BHBP at the global level. Some countries have only defined a certain BHBP for all citizens. In contrast, some countries have defined two or more BHBP to be used for different demographic groups [7, 8].

There is no unique method for prioritizing health intervention that can be ideal for all countries [9]. Selection of approaches for prioritizing intervention needs explicit and clear discussions on principles and criteria used for making decisions [10]. Developing a BHBP in many countries lacks a systemic approach and transparent criteria for decision-making process. This approach makes inefficient use of resources, while taking steps to use a clear approach for development of BHBP is an unavoidable way for all health systems [11]. Therefore, the present paper aims to use scoping review in order to extract criteria for the development of BHBP used in health systems of different countries.

## Methods

### Data resources

In order to identify relevant studies, a systematic search was carried out between January and April 2016. To assure that no other similar systematic reviews existed, a rapid search was initiated with Cochrane Library Database to find systematic articles related to the research topic. This database could not find any articles related to the study subject. Then, search was implemented in PubMed, Scopus, Science Direct, Web of Science, and ProQuest databases. Given that these databases only include published articles, WHO, World Bank, and Google websites were used to identify the most relevant reports and discussion papers and complete the scoping review. In the next stage, the reference lists of these studies were examined in order to identify the articles that were not included in the previous stage and were in line with the research objectives. Reports and articles approved by experts in this area were also included in the present study. In all search stages, the results were reviewed by another individual for the sake of reassurance.

### Search strategy

The search was implemented with relatively common terms using synonymous words and “OR” operator. To achieve more specialty; i.e., reduced non-related articles, the search was implemented with synonymous words and “AND” operator. The combination of words “title, abstract, and keyword” were used to find the related papers. The “control mesh” keyword in PubMed database was also employed to find words related to the article (Table 1).

**Table 1:** Search strategy for development of Basic Health Benefit Package

Strategy	#1 AND #2 AND #3
#1	Basic benefit package **OR** Health benefit package **OR** Benefit package **OR** Health basket **OR** Benefit basket **OR** Medical benefit package **OR** Basic Health Service Package **OR** Basic Health Insurance Package **OR** Essential benefit package **OR** Essential health care package **OR** Minimal health care package **OR** Insurance coverage **OR** Universal health coverage **OR** Universal health insurance
#2	Criteria **OR** Criterion **OR** Determinant
#3	Decision making **OR** Rationing **OR** Priority **OR** priority setting **OR** Allocation Resource **OR** MCDM **OR** Multi Criteria Decision Making **OR** Analytic Hierarchy processing (AHP) **OR** Analytic Network processing (ANP)

### Inclusion and exclusion criteria

English articles conducted on development of BHBP criteria in 1980 and later were included. However, the articles conducted before 1980, unpublished articles, and those with English abstracts published in languages other than English were excluded. Given that abundant papers were published in this area in many countries, recent articles and reports on development of BHBP criteria were included and the remaining articles published in previous years were excluded in the final stage of screening.

### Data extraction

The information required to use and combine the articles with the least credibility point were extracted using a collection and summarization form. This form included corresponding author, research population, research sample, study time, study design, data collection tools, methods, results, limitations, and conclusions. After completing the summary data form for each of the selected papers, the entire forms were synthesized item by item and the results were demonstrated in frequency descriptive tables and graphs. In the extraction stage, one of the authors inserted the data into the form and another author reexamined them. In case of contradictions between the two authors, they were resolved by discussion and exchange of views. If the debates were not settled, the view of the third author was used. These forms were planned and completed by Excel software.

### Screening

In the first stage, the entire applied articles and reports of databases and websites were reviewed by two authors (Z K, R H). In the second stage, abstracts of the selected articles and reports were reviewed by the above authors. Subsequently, full texts of the selected articles and reports were thoroughly studied and evaluated. Eventually, the articles and reports with adequate credibility that pointed to BHBP criteria were selected.

## Results

**Fig. 1:**
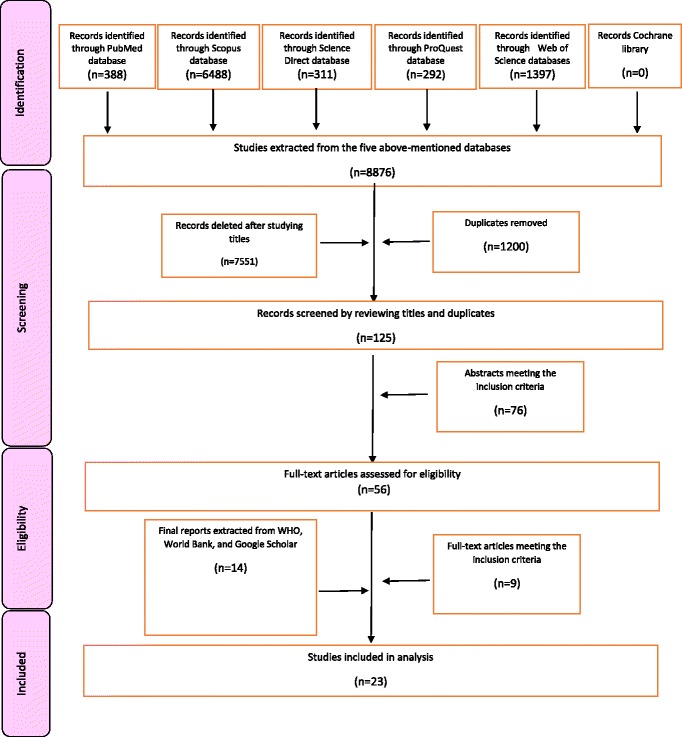
PRISMA Flow Diagram for the scoping review process

In total, 8876 articles were selected after searching the databases. Thereafter, 9 articles and 14 reports were selected for final analysis. Nevertheless, 1200 articles were excluded because of overlap in databases. By examining the remaining 7676 titles, 7551 articles were excluded due to incompatibility with the research topic. The remaining 125 articles were reviewed in terms of abstracts. Accordingly, 49 abstracts were excluded due to lack of adequate compliance with the research purpose. From the remaining 75 articles, 6 had no English texts and 14 were repetitive. Finally, 56 papers were selected to study the full texts. Among these papers, 9 had adequate credibility and explicitly pointed to development of BHBP criteria. In order to complete a comprehensive review, WHO, World Bank, and Google websites were selected to find reports and discussion papers. At this stage, 14 reports were selected for scoping review (Fig. 1).

The synthesis results are presented in Table 2. As the table depicts, 19 criteria were selected from the scoping review. Due to diversity of criteria in terms of number and nature, they were divided into three distinct categories. These categories included intervention-related, disease-related, and community-related criteria. The largest number of criteria were included in the category of intervention-related criteria. These criteria included cost-effectiveness, effectiveness, budget impact, and cost of interventions. In addition, disease-related criteria included burden of disease, severity of disease, and positive externality. Finally, community-related criteria included equity, access, affordability for the individual or household, and social values. The most widely applied criteria included cost-effectiveness (20), effectiveness (19), budget impact (12), equity (12), and burden of disease (10). On the other hand, access (2), innovation (1), and severity of disease (1) were less applied. In some countries, like the Netherlands, Germany, and England, certain criteria had no clear definitions. It should be noted that the majority of the selected articles and final reports were conducted from 2005 onwards. (Table 2).

**Table 2:** Determinant Criteria of Basic Health Benefit Package

Category	Criteria	Frequency [References]
Intervention –related criteria	▪ Cost-effectiveness ▪ Effectiveness ▪ budget impact ▪ Necessity ▪ Safety ▪ Sustainability ▪ Feasibility ▪ Costs of intervention ▪ Comprehensive ▪ Maximizing the improvement of population health status ▪ Scaling up ▪ Innovation	20 [5, 35–51] 19 [5, 35, 38–40, 43, 44, 50–55] 12 [5, 35, 36, 38, 40–42, 45–47, 49] 10 [5, 42–44, 47, 52–54] 6 [5, 40, 44, 54, 55] 5 [39, 43, 50, 53, 55] 5 [36, 37, 46, 47, 55] 4 [5, 52] 3 [36, 41, 50] 3 [40, 50] 1 [53] 1 [40]
Disease-related criteria	▪ Burden of disease ▪ Externalities ▪ Severity of disease	10 [35, 39, 41, 43, 45, 46, 49–51, 55] 2 [49, 50] 1 [35]
Community-related criteria	▪ Equity ▪ Affordability ▪ Social values ▪ Access	12 [35, 39, 40, 42, 43, 45–47, 49, 50, 53, 55] 5 [35, 37, 38, 40, 46] 4 [37, 43, 46, 48] 2 [47, 49]

## Discussion

To the best of our knowledge, this is the first scoping review on criteria for determining BHBP. These kinds of studies are conducted for preliminary assessment of potential size and scope of available research literature [12].

Moving toward UHC, health systems need to determine BHBP. The question is which criteria should be considered to develop BHBP with regard to resource constraints. In the present review, we attempted to answer this question by collecting all criteria used in different countries and categorizing them into three categories, namely intervention-related criteria, disease-related criteria, and community-related criteria. Studies conducted on inclusion criteria on new technologies and prioritization of health interventions have also used similar categories of criteria [13–16].

The findings of the present study revealed the existence of different criteria in terms of number and nature to select services priority and optimal combination of services, which might be attributed to differences in values, history, culture, and health priorities in different countries [17]. Different economic, moral, and processing approaches in prioritizing interventions and health challenges could also lead to various methods and criteria in development of BHBP [18]. As stated by WHO, every country should select a set of criteria based on its capacity to monitor the progress of UHC and data system [19]. It seems that most common criteria used in different countries have originated from two general purposes of health systems, including improving public health and equity in financial contribution.

Our study findings revealed that most of the used criteria were associated with intervention. In the intervention-related group, “cost-effectiveness” and “effectiveness” were the dominant criteria in most countries. Prioritizing services based on cost-effectiveness is the conventional method known in the world for preparing and providing the highest possible health benefits based on a certain amount of budget. Accordingly, most countries and international organizations suggest that health services must be prioritized on the basis of cost-effectiveness evidences. WHO has also recommended applying this criterion in the process of prioritizing, selecting, and expanding benefits [19].

One of the advantages of cost-effectiveness criterion is its definite definition and methodology in all countries around the world. Despite the importance of cost-effectiveness criterion, its application in the process of prioritization was limited due to political influences, community preferences, and systemic barriers such as lack of necessary data. Nevertheless, WHO has recently made access to this information possible at national level via implementation of WHO-CHOICE projects [20].

The current study findings indicated that effectiveness was another criterion frequently used in determining BHBP. At the end of the last century, we witnessed development of a clear and logical approach in prioritizing health services. One of the most important approaches was evidence-based medical development or utilization of effective interventions. This approach was founded by Cochran Co.in 1993. The effectiveness criterion was related to intervention outcomes and was commonly used in prioritizing the services [21, 22]. Another study entitled “determination of health benefits package in nine European countries” also revealed effectiveness as an important criterion [5].

According to the results, equity as a community-related criteria was used in the development of BHBP. In theoretical concepts, equity was one of the criteria and the main objective of prioritization. By improving public health condition in developing countries in the last two decades, policymakers found out health differences among different groups of people and used equity analysis for description of distributive impacts [23, 24]. The study implemented in Uganda indicated that all beneficiaries agreed to use the criterion of equity to prioritize benefits [16].

Burden of diseases might be considered as one of the first criteria used in health systems as well as in development of BHBP. Early in 1990, World Bank used this criterion to measure epidemiological burden of mortality in terms of disease burden analysis. Disease burden analysis has greatly assisted policymakers with targeted interventions in areas with high disease burden [25]. In the study by *Jayasinghe* et al., estimation of disease burden was recommended as the main prioritization approach in developing countries [26]. However, some individuals believe that burden of disease lacks a conceptual basis for prioritizing health interventions. Moreover, this criterion has been seriously criticized due to the present assumptions regarding intrinsic value and its technical limitations [27]. Nonetheless, many developing countries, like India, Kenya, Tanzania, Uganda, and Ethiopia, use burden of disease for prioritizing health benefits [28].

Necessity and cost of interventions were other criteria extracted from the results of the present study. In the comparative study performed by Stefan et al. (2005) in Germany, England, and Switzerland, necessity and cost of interventions were the criteria used for development of BHBP [29]. The concept of necessity was too obscure and broad and was not well defined [30]. However, necessity might be regarded based on evidence-orientated clinical results. In this case, it could be identical to clinical effectiveness.

The National Institute for Health and Care Excellence (NICE) in England has clearly defined the “costs of intervention” criteria in development of guidelines and evaluation of technologies [31].

In addition to cost-effectiveness, clinical effectiveness, burden of disease, necessity, and equity, several countries have focused on the impacts of inclusion costs of a service in the BHBP from the perspective of insurance organizations (budget impact) and individuals (affordability). In general, inclusion of a service into the health package involves expenses for a third payer. Therefore, impacts of inclusion costs of a service for financial strength of insurance companies should be taken into account. Furthermore, given that financial protection for households is one of the main objectives of development of BHBP, the exclusion costs of this service should be regarded. However, third payers prefer to select services with lower costs. On the contrary, lack of coverage of interventions leading to catastrophic health expenditure or impoverishment will result in lower effectiveness of BHBP in moving toward UHC.

The results of prior studies have indicated comprehensive of services and severity of disease as the criteria that should be considered in the development of BHBP. The important point is widespread improvement of services and paying attention to all areas of service provision from promotion to rehabilitation. Countries, such as India, Kenya, and the Philippines, that initially covered only inpatient services, are now moving towards development of advantages to cover primary and preventive services. This might be due to recognizing the fact that although outpatient services may be costly, they may lead to greater impacts on health outcomes [32].

The criterion of severity of disease is broadly applied in prioritizing health services to balance equity and efficiency [30, 33]. The study by Makundi et al. used severity of disease as one of the criteria for development and prioritization of BHBP [34].

It is important to note that decision-making and prioritization are dynamic processes just as interventions that are not currently allowed to enter the package may be prioritized with passage of time. For example, changes in population structure may result in reduced or increased prevalence of some diseases or access to new technologies may lead to changes in existing expenses [35].

## Limitations

Like other studies, the present research had certain limitations. It only included papers and reports published in English, and gray resources (unpublished reports and proceedings of conferences) were excluded.

## Conclusion

It is evident that existence of a logical scientific model for designing health benefits at different levels of the health system could lead to more appropriate allocation and management of available health resources and consideration of needs and priorities as the key criterion in health policymakers’ decision making. Prerequisites and requirements for entry into this arena include promoting and strengthening scientific promotion regarding decision-making capacities and evidence-based policymaking, which depends on existence of a credible information system and determination of local priorities based on needs [32].

The findings of the present study suggested that although criteria and processes of BHBP development were different depending on economic, social, and cultural conditions and country-specific values, with regard to the frequency of using certain criteria in countries under study, several criteria such as cost-effectiveness, effectiveness, burden of disease, equity, necessity, and budget impacts were recommended. Future studies on assessment of success of countries with different types of criteria for BHBP will help policymakers to choose appropriate criteria.

## Abbreviation

BHBP: Basic Health Benefit Package
UHC: Universal Health Coverage
WHO: World Health Organization
